# Unique pattern of endometrial invasion in gastric-type adenocarcinoma of the uterine cervix: a report of two cases

**DOI:** 10.1007/s13691-026-00858-2

**Published:** 2026-03-12

**Authors:** Kyosuke Kamijo, Tsutomu Miyamoto, Hirofumi Ando, Hisanori Kobara, Yayoi Satoh, Yaeko Kobayashi, Tanri Shiozawa

**Affiliations:** 1 Department of Obstetrics and Gynecology, Shinshu University School of Medicine, 3-1-1 Asahi, Matsumoto, 390-8621 Japan; 2 Department of Obstetrics and Gynecology, Nagano Prefectural Shinshu Medical Center, 1332 Suzaka, Suzaka, 382-8577 Japan; 3 Department of Laboratory Medicine, Shinshu University School of Medicine, 3-1-1 Asahi, Matsumoto, 390-8621 Japan; 4 Department of Diagnostic Pathology, Shinonoi General Hospital, 666-1 Shinonoiai, Nagano, 388-8004 Japan; 5 Department of Gynecology, Nagano Municipal Hospital, 1333-1 Tomitake, Nagano, 381-8551 Japan

**Keywords:** Cervical cancer, Endometrial invasion pattern, Gastric-type adenocarcinoma, Human papillomavirus-independent, Radical hysterectomy

## Abstract

Gastric-type cervical adenocarcinoma (GAS), the most prevalent subtype of human papillomavirus (HPV)-independent cervical adenocarcinoma, is an aggressive malignancy with a poor prognosis. We herein present two cases of GAS with a unique endometrial infiltration pattern. Both cases were 37-year-old nulligravid women presenting with advanced GAS. A pathological examination revealed HPV-independent GAS that was positive for claudin 18 and negative for p16, with extensive invasion, including the myometrium and endometrium. Endometrial infiltration was characterized by a distinctive “symbiotic” pattern of invasion. In these areas, GAS glands intermingled with normal endometrial glands without disrupting the native architecture, and there was no distinct tumor border or stromal reaction. An immunohistochemical analysis revealed fewer CD8-positive tumor-infiltrating lymphocytes (TILs) around invasive GAS glands in the endometrium than in the normal endometrium and both the tumor center and invasive margin of the primary cervical lesion. These results are consistent with relative T-cell exclusion at the tumor–endometrium interface. This “symbiotic” invasion pattern differs from typical cervical adenocarcinoma, which forms distinct boundaries with desmoplastic stromal reactions. The observed pattern may contribute to the unexpectedly extensive spread of GAS frequently discovered only after surgical resection. The reduction in CD8-positive TILs density around invasive GAS glands indicates an immunologically “cold” tumor microenvironment that may contribute to treatment resistance. The present results provide novel insights into the pathology of GAS that may inform more effective diagnostic approaches and therapeutic strategies for this aggressive malignancy.

## Introduction

Gastric-type cervical adenocarcinoma (GAS) is an aggressive malignancy and the most prevalent subtype among human papillomavirus (HPV)-independent cervical adenocarcinomas classified in the World Health Organization 2020 classification [1, 2]. GAS is characterized by a poor prognosis and resistance to conventional chemotherapy and radiotherapy regimens [2, 3].

Unlike typical cervical adenocarcinoma that forms an apparent mass with a well-defined tumor boundary [4, 5], GAS frequently forms an ill-defined mass and spreads extensively with a low tumor cell density; therefore, the tumor boundary of GAS is often ambiguous [6]. This invasion pattern makes it challenging to detect the extent of tumor spread before and during surgery, and GAS frequently spreads more extensively than expected. We previously described this pattern as “stealth” infiltration, in which a minimal stromal reaction at the invasive front may obscure the boundary and contribute to positive surgical margins [6]. However, the relationship of this pattern with the immunological characteristics of GAS, particularly its unique infiltration mechanism, remains unclear.

Tumor-infiltrating lymphocytes (TILs), particularly CD8-positive cytotoxic T cells, are a tissue-level readout of anti-tumor immunity and have been associated with favorable outcomes in cervical cancer [7]. Since immune infiltration may be spatially heterogeneous, immune scoring approaches quantify T cells in prespecified regions, including the tumor center and invasive margin [8].

We herein describe two cases of GAS with a distinctive “symbiotic” pattern of endometrial involvement, in which tumor glands intermingled with normal endometrial glands without an obvious invasive front or stromal reaction. To investigate the relationship between this infiltration pattern and the immune system, we assessed CD8-positive TILs at the tumor–endometrium interface and, to provide regional context, compared these findings with the normal endometrium and with both the tumor center and invasive margin of the primary cervical lesion.

## Case report

### Case 1

A 37-year-old nulligravid woman presented with a watery vaginal discharge. Cervical biopsy confirmed GAS. A pelvic examination suggested vaginal and parametrial involvement. Magnetic resonance imaging (MRI) revealed an ill-defined 3-cm cervical lesion with anterior vaginal wall thickening, while computed tomography (CT) showed no distant metastasis or significant lymphadenopathy. Serum tumor markers, including carcinoembryonic antigen (CEA), carbohydrate antigen 125 (CA125), and carbohydrate antigen 19-9 (CA19-9), were within normal ranges. The patient was diagnosed with International Federation of Gynecology and Obstetrics (FIGO) 2018 clinical stage IIIA disease. The patient was premenopausal, and surgery was performed in the proliferative phase. The patient underwent abdominal radical hysterectomy with bilateral salpingo-oophorectomy and total vaginectomy. Intraoperative peritoneal cytology was negative.

**Fig. 1:**
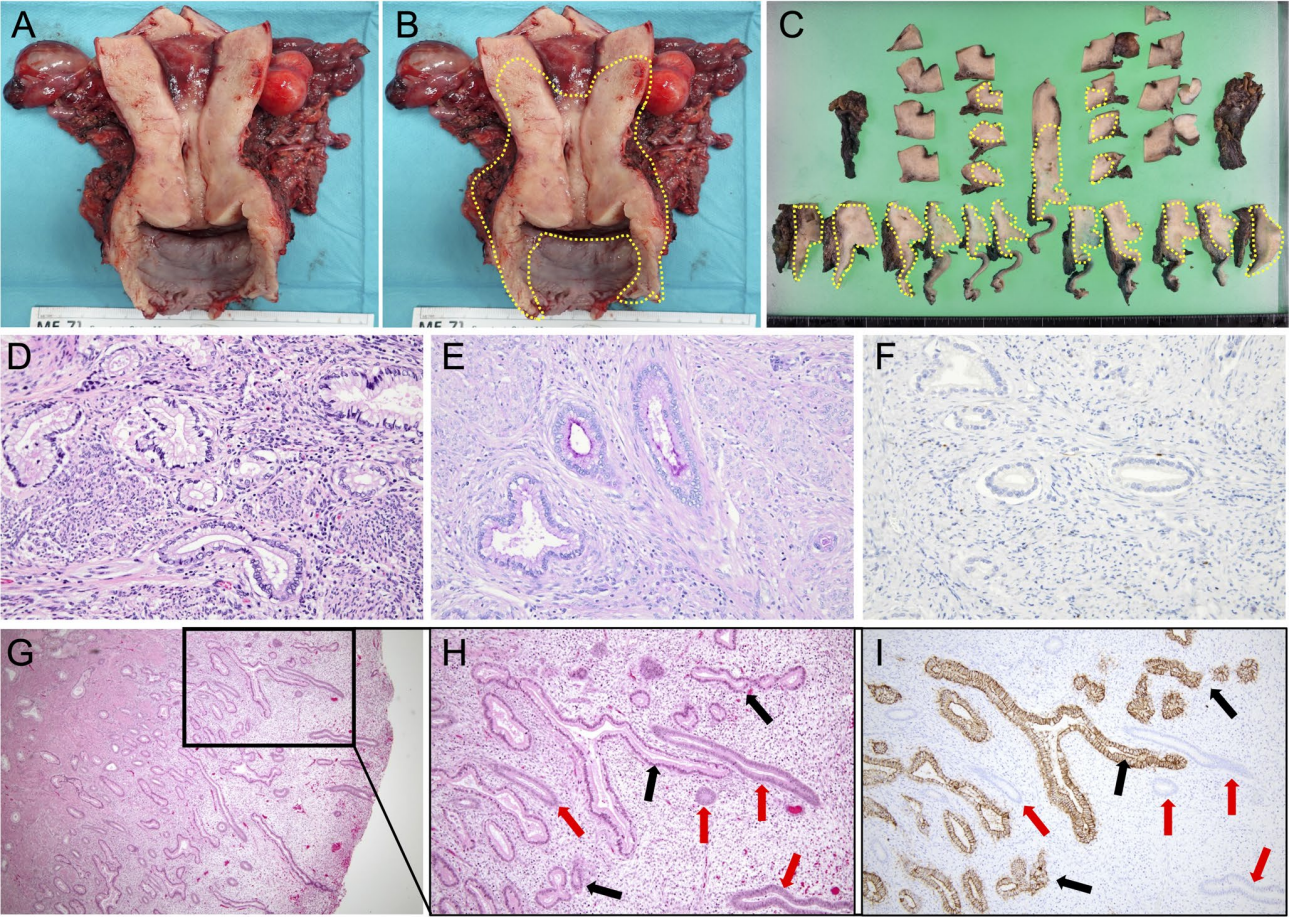
Gross and histopathological features of gastric-type cervical adenocarcinoma (GAS) in Case 1. (**A**, **B**) Gross findings of the radical hysterectomy specimen opened by an anterior longitudinal incision through the cervix and vagina. (**A**) The endocervical and endometrial surfaces appear smooth without a distinct mass. (**B**) The area outlined by the yellow dashed line indicates the extent of histologically confirmed tumor involvement: GAS diffusely infiltrates the entire cervix and extends to the lower uterine corpus, vaginal wall, and parametrium. (**C**) Twelve serial transverse sections of the resected uterus; the yellow dashed line denotes the distribution of the microscopic tumor. (**D**–**F**) Representative microscopic findings of GAS in the cervix. (**D**) Hematoxylin and eosin (HE) staining (200×) shows the infiltrative proliferation of irregular glands lined by mucinous columnar cells with an abundant pale-to-clear cytoplasm and variable nuclear atypia, consistent with GAS. (**E**) Alcian blue/Periodic acid–Schiff (AB/PAS) staining (200×) shows mainly PAS-positive (neutral) intracellular mucin with weak Alcian blue staining, highlighted as magenta/red. (**F**) Immunohistochemistry for p16 (200×) was negative in tumor glands. (**G**–**I**) The endometrium infiltrated by GAS glands. (**G**) HE staining (40×) shows GAS glands infiltrating among normal endometrial glands and stroma; the black boxed area is shown at a higher magnification in (**H**) and (**I**). (**H**) HE staining (100×) and (**I**) claudin 18 (CLDN18) immunohistochemistry (100×) of serial sections show CLDN18-positive GAS glands (black arrows) interspersed between CLDN18-negative normal endometrial glands (red arrows) without any stromal reaction

A macroscopic examination of the hysterectomy specimen showed anterior vaginal wall thickening without a discrete cervical mass (Fig. 1A). Histologically, gastric-type cervical adenocarcinoma diffusely involved the entire cervix and extended to the lower uterine corpus, vaginal wall, and parametrium, as illustrated by serial section mapping (Fig. 1B–C). The histological tumor size was 90 mm, with full-thickness cervical stromal invasion (20/20 mm), lymphovascular space invasion, and pelvic lymph node metastasis. Hematoxylin and eosin (HE) staining demonstrated infiltrative irregular glands lined by mucinous columnar cells with a pale-to-clear cytoplasm and variable nuclear atypia (Fig. 1D). Alcian blue/Periodic acid–Schiff (AB/PAS) staining showed predominantly PAS-positive (Alcian blue–negative) intracellular mucin (Fig. 1E). Tumor glands were positive for gastric markers including HIK1083 and claudin 18 (CLDN18), whereas p16 was negative (Fig. 1F). The final pathological diagnosis was HPV-independent adenocarcinoma, GAS, pT3aN1M0 (UICC 2021), pathological stage IIIC1 (FIGO 2018). The anterior vaginal wall margin was positive. Two months later, a refractory urethral fistula developed, and recurrence was suspected (posterior bladder wall thickening with bilateral inguinal lymphadenopathy); therefore, the patient underwent total cystourethrectomy with ureterocutaneostomy, partial omentectomy, and bilateral inguinal lymphadenectomy. Pathology confirmed GAS in the omentum only. The patient subsequently received whole-pelvic irradiation and remained free of recurrence during the follow-up. Two years after the initial surgery, the patient was transferred for the management of refractory intestinal obstruction.

**Fig. 2:**
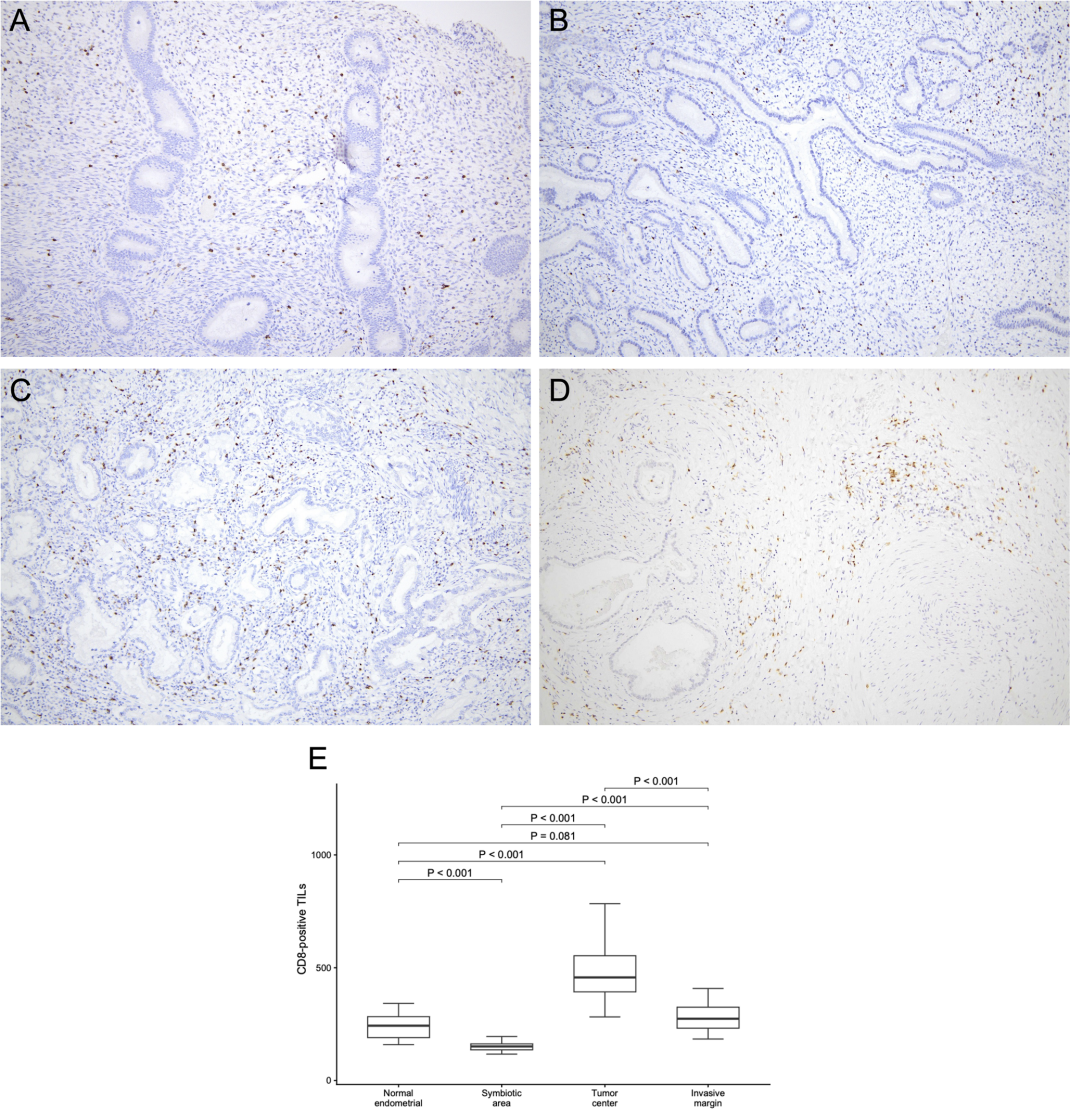
Comparison of CD8-positive tumor-infiltrating lymphocytes (TILs) across four regions in Case 1. (**A**–**D**) Representative CD8 immunohistochemistry (100×) illustrating the distribution of CD8-positive TILs in (**A**) the normal endometrium (density, 177 cells/mm^2^), (**B**) the “symbiotic” area (135 cells/mm^2^), (**C**) the tumor center (533 cells/mm^2^), and (**D**) the invasive margin (312 cells/mm^2^). (**E**) Box-and-whisker plots comparing the density of CD8-positive TILs quantified in 15 hotspot fields per region. Boxes indicate the interquartile range, with the median shown as a horizontal line; whiskers extend to the most extreme values within 1.5× the interquartile range, and dots represent outliers beyond this range. Pairwise comparisons were performed using the Mann–Whitney U test, with the Bonferroni correction for multiple comparisons (Bonferroni-adjusted significance threshold, *P* < 0.0083 [0.05/6]). The “symbiotic” area showed a significantly lower density of CD8-positive TILs than the other regions (all *P* < 0.001)

**Fig. 3:**
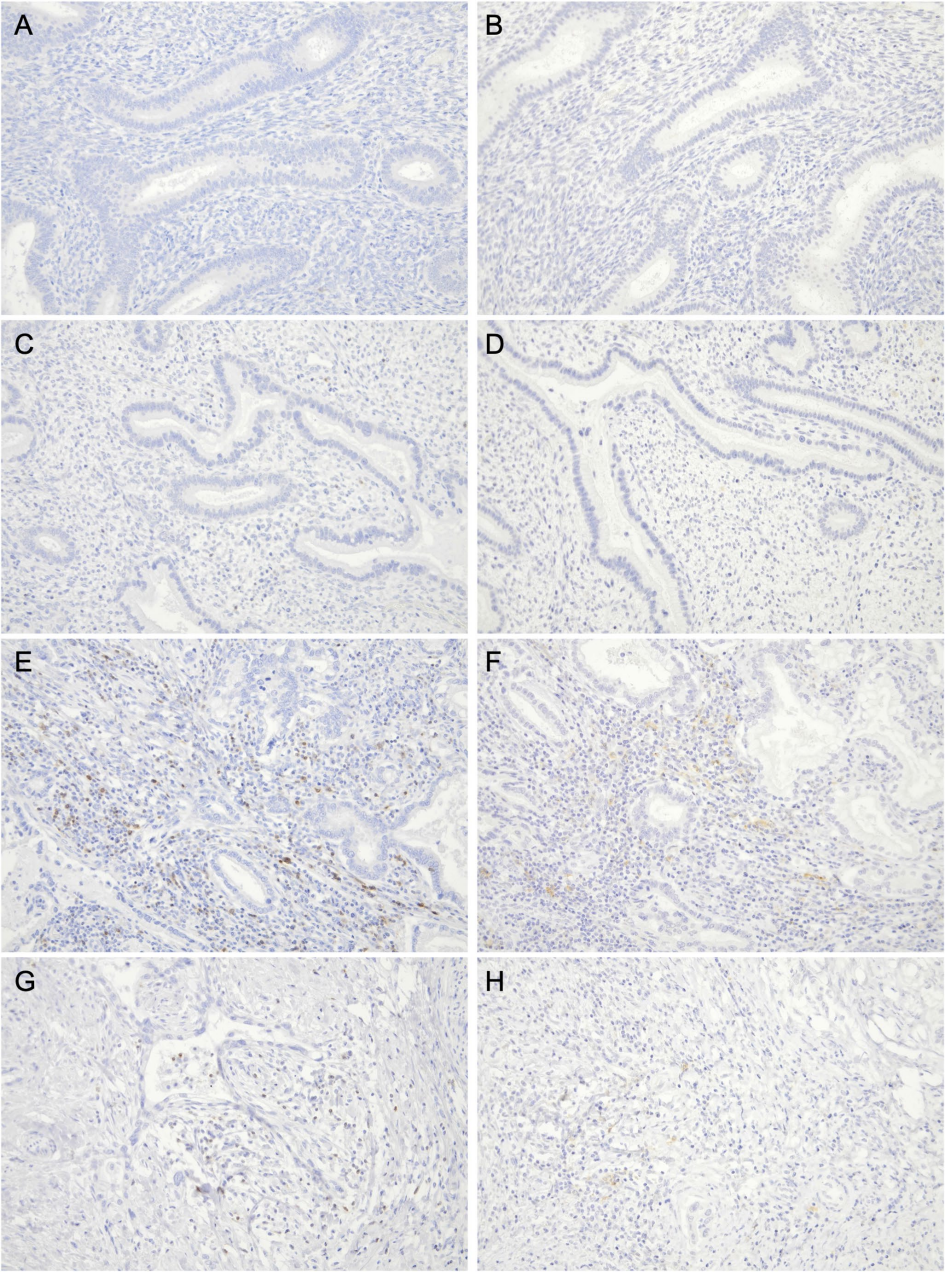
Immunohistochemical staining of programmed cell death protein 1 (PD-1) and programmed death-ligand 1 (PD-L1) across different regions in Case 1. All panels are shown at 200×. (**A**, **B**) The normal endometrium stained for (**A**) PD-1 and (**B**) PD-L1; combined positive score (CPS) = 0. (**C**, **D**) The “symbiotic” area stained for (**C**) PD-1 and (**D**) PD-L1; CPS = 0. (**E**, **F**) The tumor center stained for (**E**) PD-1 and (**F**) PD-L1; CPS > 10. (**G**, **H**) The invasive margin stained for (**G**) PD-1 and (**H**) PD-L1; CPS = 4

The morphology of the background endometrium was consistent with the proliferative phase on HE staining (Fig. 1G–H), indicating that “symbiotic” glands were intermingled predominantly within the functional layer. Microscopically, CLDN18-positive GAS glands infiltrated from the myometrium into the endometrium and intermingled with CLDN18-negative normal endometrial glands without disrupting the endometrial structure or any stromal reaction (Fig. 1I). Figure 2A–D shows representative CD8 immunohistochemistry for the four regions. We quantified the density of CD8-positive TILs at 100× magnification (cells/mm^2^) in 15 hotspot fields per region (*n* = 15). The endometrial “symbiotic” invasion area showed a lower CD8-positive TILs density (median 151 [interquartile range(IQR) 136.0–162.5]) than the normal endometrium (median 243 [190.0–283.0]), the tumor center of the primary cervical lesion (median 457 [393.0–553.0]), or the invasive margin of the primary cervical lesion (median 274 [232.0–325.0]) (*P* < 0.001 for all pairwise comparisons after the Bonferroni correction; Fig. 2E). Programmed cell death protein 1 (PD-1) immunostaining revealed PD-1–positive immune cells, and programmed death-ligand 1 (PD-L1) expression assessed by the combined positive score (CPS) was 0 in the “symbiotic” area and normal endometrium, > 10 in the tumor center, and 4 at the invasive margin (Fig. 3A–H).

### Case 2

A 37-year-old nulligravid woman with no history of sexual intercourse presented with abnormal genital bleeding. Cervical biopsy confirmed adenocarcinoma with a negative high-risk HPV test. A pelvic examination suggested right parametrial involvement. CA19-9 was markedly elevated (2651.8 U/mL), whereas CEA and CA125 were not. MRI revealed an ill-defined 4-cm cervical tumor, and CT showed no distant metastasis. The patient was staged as FIGO 2018 clinical IIB and underwent abdominal radical hysterectomy with bilateral salpingo-oophorectomy. Intraoperative peritoneal cytology was positive.

**Fig. 4:**
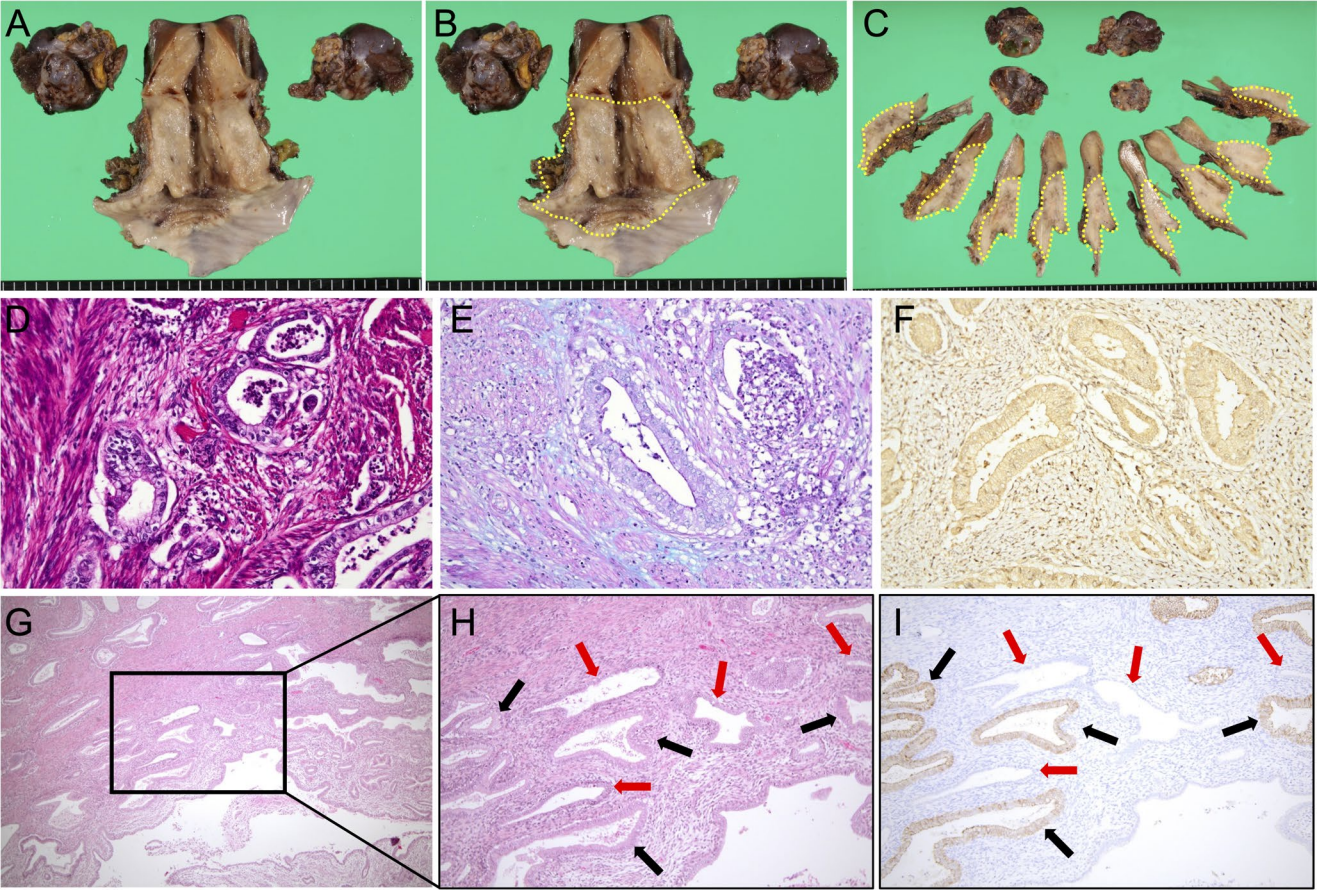
Gross and histopathological features of gastric-type cervical adenocarcinoma (GAS) in Case 2. (**A**, **B**) Gross photograph of the hysterectomy specimen opened by an anterior longitudinal incision through the cervix and vagina. (**A**) A nodular lesion is present in the cervix, whereas the endometrial surface appears smooth without an obvious exophytic mass. (**B**) The yellow dashed line indicates the distribution of the histologically confirmed tumor: GAS diffusely involves the cervix and extends into the uterine corpus, vaginal wall, and parametrium. (**C**) Twelve serial transverse sections of the resected uterus; the yellow dashed line denotes the distribution of microscopic tumor involvement. (**D**–**F**) Representative microscopic findings of GAS in the cervix. (**D**) Hematoxylin and eosin (HE) staining (200×) shows the infiltrative proliferation of irregular glands lined by mucinous columnar cells with an abundant pale-to-clear cytoplasm and variable nuclear atypia, consistent with GAS. (**E**) Alcian blue/Periodic acid–Schiff (AB/PAS) staining (200×) shows mainly PAS-positive (neutral) intracellular mucin with weak Alcian blue staining, highlighted as magenta/red. (**F**) Immunohistochemistry for p16 (200×) was negative (non–block-type) in tumor glands. (**G**–**I**) The endometrium infiltrated by GAS glands. (**G**) HE staining (40×) shows GAS glands infiltrating among normal endometrial glands and stroma; the black boxed area is shown at a higher magnification in (**H**) and (**I**). (**H**) HE staining (100×) and (**I**) claudin 18 (CLDN18) immunohistochemistry (100×) of serial sections show CLDN18-positive GAS glands (black arrows) interspersed between CLDN18-negative normal endometrial glands (red arrows) without an associated stromal reaction

Macroscopically, the hysterectomy specimen showed a nodular cervical lesion (Fig. 4A), and the distribution of the histologically confirmed tumor is outlined by the yellow dashed line in Fig. 4B. Serial transverse sections revealed diffuse microscopic tumor involvement extending from the cervix into the uterine corpus, vaginal wall, and parametrium (Fig. 4C). The histological tumor size was 65 mm, with full-thickness cervical stromal invasion (19/19 mm), lymphovascular space invasion, and pelvic lymph node metastasis. HE staining showed infiltrative irregular glands (Fig. 4D), and mainly PAS-positive intracellular mucin was observed with AB/PAS staining (Fig. 4E). Tumor glands were diffusely positive for CLDN18 and negative for p16 (Fig. 4F). The final pathological diagnosis was HPV-independent adenocarcinoma, GAS, pT2bN1M0 (UICC 2021), pathological stage IIIC1 (FIGO 2018). The patient received adjuvant concurrent chemoradiation with whole-pelvic irradiation and platinum-based chemotherapy and has remained free of disease for five years.

**Fig. 5:**
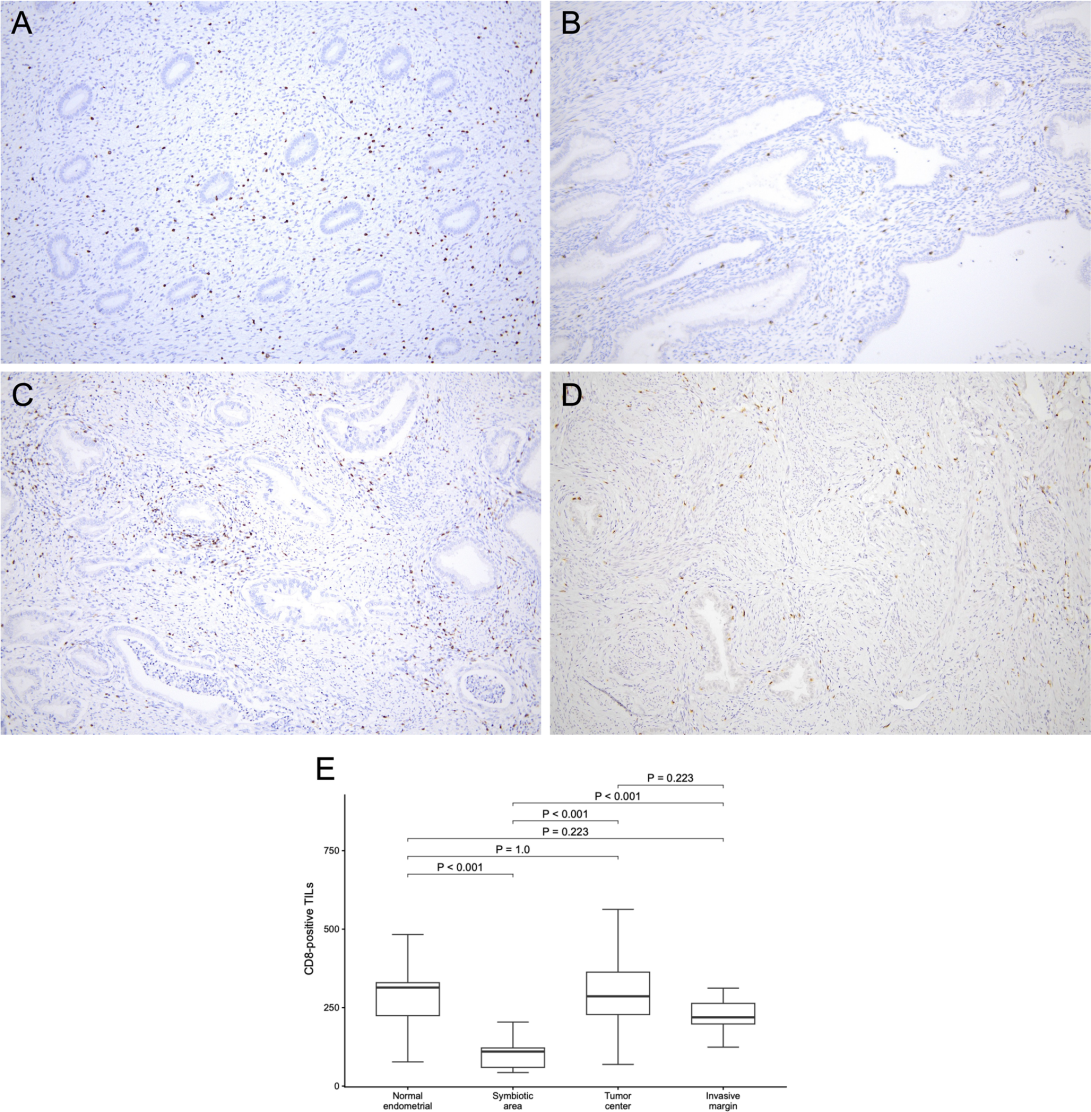
Comparison of CD8-positive tumor-infiltrating lymphocytes (TILs) across four regions in Case 2. (**A**–**D**) Representative CD8 immunohistochemistry (100×) illustrating the distribution of CD8-positive TILs in (**A**) the normal endometrium (density, 161 cells/mm^2^), (**B**) the “symbiotic” area (117 cells/mm^2^), (**C**) the tumor center (282 cells/mm^2^), and (**D**) the invasive margin (232 cells/mm^2^). (**E**) Box-and-whisker plots comparing the density of CD8-positive TILs quantified in 15 hotspot fields per region. Boxes indicate the interquartile range, with the median shown as a horizontal line; whiskers extend to the most extreme values within 1.5× the interquartile range, and dots represent outliers beyond this range. Pairwise comparisons were performed using the Mann–Whitney U test, with the Bonferroni correction for multiple comparisons (Bonferroni-adjusted significance threshold, *P* < 0.0083 [0.05/6]). The “symbiotic” area demonstrated a significantly lower density of CD8-positive TILs than the other regions (all *P* < 0.001)

**Fig. 6:**
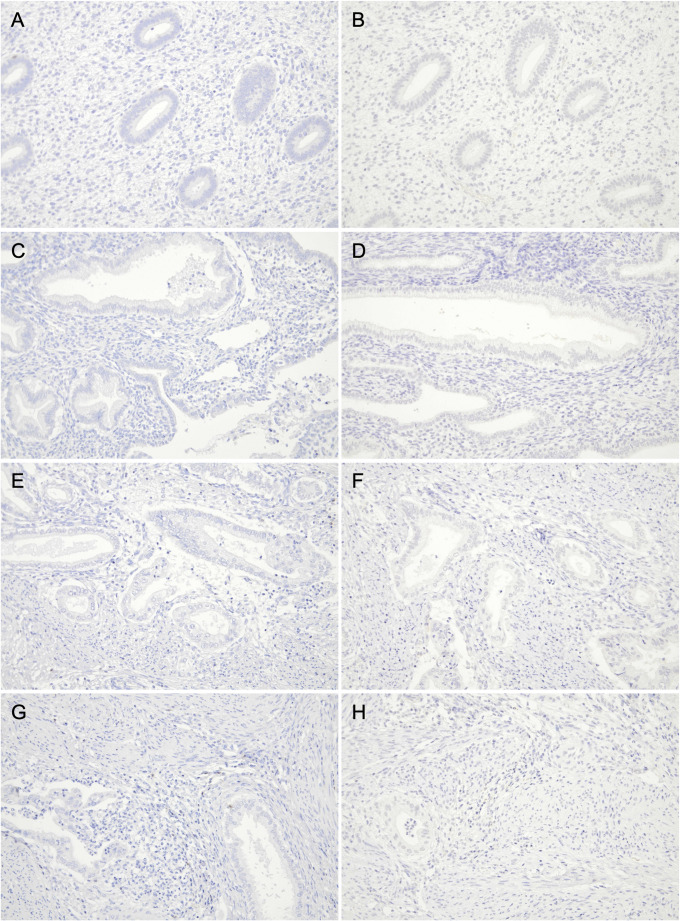
Immunohistochemical staining of programmed cell death protein 1 (PD-1) and programmed death-ligand 1 (PD-L1) across different regions in Case 2. All panels are shown at 200×. (**A**, **B**) The normal endometrium stained for (**A**) PD-1 and (**B**) PD-L1; combined positive score (CPS) = 0. (**C**, **D**) The “symbiotic” area stained for (**C**) PD-1 and (**D**) PD-L1; CPS = 0. (**E**, **F**) The tumor center stained for (**E**) PD-1 and (**F**) PD-L1; CPS = 0. (**G**, **H**) The invasive margin stained for (**G**) PD-1 and (**H**) PD-L1; CPS = 0

Similar to Case 1, the morphology of the background endometrium was consistent with the proliferative phase on HE staining (Fig. 4G–H), supporting “symbiotic” invasion being the most evident in the functional layer. Microscopically, CLDN18-positive GAS glands infiltrated from the myometrium into the endometrium and intermingled with CLDN18-negative normal endometrial glands without disrupting the endometrial structure or any stromal reaction (Fig. 4I). Using the same hotspot-based approach as in Case 1 (15 fields/region at 100×; Fig. 5A–D), CD8-positive TILs density (cells/mm^2^) was lower in the endometrial “symbiotic” invasion area (median 110 [IQR 59.5–121.5]) than in the normal endometrium (median 314 [224.5–329.5]), the tumor center of the primary cervical lesion (median 286 [228.0–363.0]), and the invasive margin of the primary cervical lesion (median 219 [198.0–263.5]) (all *P* < 0.001; the Mann–Whitney U test with the Bonferroni correction; Fig. 5E). PD-1 immunostaining showed no PD-1–positive immune cells, and PD-L1 expression assessed by CPS was 0 in all four regions (Fig. 6A–H).

## Discussion

The present cases highlight two key pathological findings relevant to the diagnosis and treatment of GAS. GAS may exhibit a distinctive “symbiotic” pattern of endometrial infiltration, in which tumor cells intermingle with normal endometrial components without forming well-defined borders, stromal reactions, or disrupting the native glandular architecture, making GAS glands appear similar to normal endometrial glands. Furthermore, within this “symbiotic” area, the density of CD8-positive TILs was lower than in the normal endometrium and primary cervical lesion, and PD-1/PD-L1 staining provided additional regional context.

The unique “symbiotic” endometrial invasion pattern in our two cases is consistent with a characteristic invasion pattern of GAS in which the tumor–normal interface may not be clearly defined. Cervical adenocarcinomas typically show destructive stromal invasion with desmoplastic stromal reactions or fibrosis that help delineate tumor margins [4, 5, 9]. In contrast, we previously reported that GAS often lacked a macroscopically apparent tumor mass and instead infiltrated diffusely with a low tumor cell density, making the preoperative and intraoperative identification of the tumor extent challenging (“stealth” infiltration) [6]. We further noted that sparse invasion fronts in GAS may show a minimal stromal reaction, which may obscure the invasion fronts. In the present cases, apparent stromal reactions were detected in the center of the primary cervical regions, and GAS exhibited markedly different behavior at the invasion fronts, particularly at the endometrium. The characteristic “symbiotic” endometrial invasion pattern was most apparent in the endometrial functional layer; since this layer undergoes cyclical proliferation, shedding, and repair, we speculate that cyclical endometrial remodeling may facilitate this pattern. Superficially intermingled GAS glands without a stromal reaction may even be shed with the functional layer during menstruation, potentially contributing to the unexpectedly extensive spread recognized in hysterectomy specimens.

Furthermore, the reduction in CD8-positive TILs around GAS glands in “symbiotic” endometrial invasion areas suggests relative T-cell exclusion at the site of invasion. CD8-positive TILs play a key role in anti-tumor immunity and are associated with an improved prognosis in various malignancies [10]. Immune infiltration in the primary cervical tumor is generally higher in HPV-associated cervical adenocarcinoma than in HPV-independent tumors, whereas data on the region-specific immune architecture in GAS remain limited [7, 11]. Although the precise mechanism by which GAS escapes immune surveillance remains unclear, immune evasion may partly explain its resistance to standard chemotherapy and concurrent chemoradiotherapy [2, 3]. Additionally, the effectiveness of immune checkpoint inhibitors (ICIs), such as anti-PD-1/PD-L1 therapies, is linked to a high TILs density and the presence of a “hot” tumor microenvironment [12]. It remains unclear whether GAS represents an “immune-cold” phenotype or if this affects the efficacy of ICIs; however, the present results suggest that the tumor–endometrium interface is “cold”, a concept that has been reported as a barrier to immunotherapy in diffuse-type gastric cancer [13].

The reduced density of CD8-positive TILs around GAS glands in “symbiotic” endometrial invasion areas may be the most apparent in the functional layer, which undergoes cyclical shedding and repair and shows marked, phase-dependent changes in the composition of leukocytes. In this setting, GAS glands that intermingle with normal endometrial glands without a stromal reaction may generate weaker inflammatory and chemotactic signals for CD8-positive T-cell recruitment, and some glands may be shed with the functional layer during menstruation, limiting the sustained accumulation of adaptive immune cells at the invasion interface [14, 15].

In Case 1, PD-L1 CPS was 0 at the “symbiotic” area and in the normal endometrium, but was higher in the tumor center and present at the invasive margin, whereas PD-1/PD-L1 staining in Case 2 was negative across all four regions. Collectively, these results suggest that immune features vary by region within GAS. However, we did not evaluate additional immune subsets, and we cannot infer mechanisms or predict responses to ICIs from two cases.

In summary, the present cases each had a characteristic “symbiotic” invasion pattern of GAS, infiltrating among normal endometrial glands without an apparent tumor boundary or stromal reaction. Additionally, the reduced density of CD8-positive TILs at the tumor–endometrium interface suggests relative T-cell exclusion at the site of invasion. Therefore, GAS glands may grow among normal endometrial glands without detection by the immune system. The present results provide novel insights into its pathological features and may contribute to the development of more effective therapeutic strategies for this aggressive malignancy. Future studies are needed to confirm the present results using a standardized spatial immune assessment and broader immune profiling, and also to elucidate the relationships between these patterns and treatment responses and outcomes.

## Data Availability

The data are not publicly available due to privacy or ethical restrictions.
